# Supplementary material for: Determinants of COVID-19 Vaccinations among a State-Wide Year-Long Surveillance Initiative in a Conservative Southern State
Source: Vaccines (Basel). 2022 Mar 9;10(3):412. doi: 10.3390/vaccines10030412 (PMC8949372; doi:10.3390/vaccines10030412)
Supplement: Supplementary file 1 [file vaccines-10-00412-s001.zip › vaccines-1605988-supplementary.pdf]

Supplementary Material

Table S1: Assessment of the factor-loading matrix of principal components

Variables	Principal Component 1	Principal Component 2	Principal Component 3	Principal Component 4
**Trust**				
**Thinks vaccines are safe**	**-0.62**	**0.40**	0.03	-0.21
**Thinks vaccines are effective**	**-0.61**	**0.40**	0.02	-0.21
**Trust in pharmaceutical research behind the vaccines**	**-0.60**	**0.50**	0.04	-0.13
**Trust in FDA approval process of the vaccine**	**-0.57**	**0.49**	0.04	-0.07
**Trust in government being forthcoming**	0.28	**0.43**	0.22	**0.43**
**Trust in the government telling the truth**	0.28	**0.49**	0.30	**0.48**
**Thinks doctors have the best interests in patients when it comes to COVID-19**	-0.23	0.21	0.12	-0.22
**Trust of information from the government on COVID-19**	0.27	**0.52**	0.28	**0.43**
**Trust in healthcare workers when it comes to COVID-19**	0.12	**0.52**	0.31	-0.18
**Preventative efforts**				
**Social distancing**	**-0.40**	-0.39	-0.09	0.20
**Wearing a mask**	**-0.40**	-0.33	-0.07	0.25
**Self-Isolation**	0.32	-0.30	-0.08	0.22
**Concern**				
**Concern in someone in the household contracting COVID-19**	-0.24	-0.34	-0.11	0.38
**Concern of the spread of COVID-19 within the community**	-0.30	0.01	-0.05	**0.47**
**Environment**				
**Frontline medical worker**	0.35	-0.35	**0.49**	-0.17

**Essential worker**	0.37	**-0.41**	0.26	-0.23
**Source of information regarding the COVID-19 pandemic**				
**Health Professionals**	**0.51**	0.23	0.08	-0.19
**News**	**0.53**	0.27	-0.35	-0.05
**Public health officials**	**0.61**	0.38	-0.16	0.08
**Government officials**	**0.65**	0.28	-0.30	0.07
**Television**	**0.46**	0.39	**-0.46**	-0.06
**Social media**	**0.60**	-0.10	-0.39	-0.13
**Friends, family, neighbors**	**0.53**	-0.03	-0.40	-0.18
**Federal briefings**	**0.58**	**0.45**	-0.20	0.28
**Other**	0.28	-0.36	0.25	-0.02
**Feelings of stress or depression**				
**Feeling sad, lonely, or depressed**	0.14	-0.35	**-0.45**	-0.04
**Feeling stressed**	0.21	**-0.44**	-0.34	-0.06
**Demographics**				
**Ethnicity**				
**White**	0.14	0.18	-0.23	**-0.49**
**Hispanic**	0.39	0.18	0.15	0.15
**Black**	**0.40**	-0.20	0.27	0.13
**Asian**	0.29	-0.09	0.08	0.30
**Other**	0.34	-0.20	0.16	0.29
**Gender**				
**Male/Female**	0.31	0.10	0.37	-0.11
**Other**	0.29	-0.20	0.27	0.08
**Income**	-0.27	**-0.41**	0.04	0.06
**Age**	-0.03	0.05	**-0.61**	-0.04
**Having Health Problems**	0.31	0.15	0.27	-0.23

Note: Factor loadings greater than 0.40 in absolute value are highlighted in bold

Table S2. Log-rank and trend tests for variables of interest

Variable*	Log-Rank Test			Multiple Comparisons		
	Chi-Square	DF	Pr>Chi-Square	Strata	Chi-Square	Bonferroni p-value
**Age**	1667.27	2	<.0001	<18-29 vs. 30-59	467.1	<.0001
				<18-20 vs. 60-70+	1534.9	<.0001
				30-59 vs. 60-70+	1442.9	<.0001
**Gender**	5.92	1	0.01	-	-	-
**Income**	32.58	3	<.0001	<$34,999 vs. $35K-$74,999	2.30	0.7
				<$34,999 vs. $75K-$100K	23.37	<.0001
				<$34,999 vs. Prefer not to answer	15.52	0.0005
				$35K-$74,999 vs. $75K-100K	8.64	0.02
				$35K-$74,999 vs. Prefer not to answer	4.05	0.3
				$75K-100K vs. Prefer not to answer	1.50	1.0
**Race**	64.46	4	<.0001	White vs. Black	31.58	<.0001
				White vs. Asian	41.38	<.0001
				White vs. Hispanic	48.73	<.0001
				White vs. Other	60.51	<.0001
				Black vs. Asian	2.45	1.0
				Black vs. Hispanic	0.08	1.0
				Black vs. Other	1.72	1.0
				Asian vs. Hispanic	3.22	0.7
				Asian vs. Other	15.50	0.0008
				Hispanic vs. Other	4.52	0.3
**Comorbidities**	164.17	1	<.0001	-	-	-
**Ever tested positive for COVID-19**	388.50	1	<.0001	-	-	-
**Thinks vaccines are safe**	2710.68	2	<.0001	No vs. Yes	2598.4	<.0001
				No vs. Not sure	299.0	<.0001
				Yes vs. Not sure	2097.1	<.0001
**Thinks vaccines are effective**	2039.62	2	<.0001	No vs. Yes	1991.4	<.0001
				No vs. Not sure	289.9	<.0001
				Yes vs. Not sure	1579.5	<.0001
**Trusts the pharmaceutical research behind vaccines**	2423.55	2	<.0001	No vs. Yes	2366.5	<.0001
				No vs. Not sure	15.06	<.0001
				Yes vs. Not sure	1595.5	<.0001

**Got the vaccine to protect family or friend at high-risk for severe disease**	1011.77	1	<.0001	-	-	-
**Got the vaccine to protect self**	2546.98	1	<.0001	-	-	-
**Got the vaccine to help control the pandemic**	1938.99	1	<.0001	-	-	-
**Frontline medical worker**	186.30	1	<.0001	-	-	-
**Thinks doctors have the best interest in patients when it comes to COVID-19**	418.65	2	<.0001	No vs. Yes	134.5	<.0001
				No vs. Not sure	237.9	<.0001
				Yes vs. Not sure	398.0	<.0001

Table S3. Health survey questions and responses.

Section 1: Let's get started		
**No.**	**Question**	**Response**
1.	What is your unique project ID?<br>*(Psst. It's on the back of your invitation letter)*	
2.	What is your home zip code?	
3.	What is your last name?	
4.	What is today's date?	M M D D Y Y Y Y
**Section 2: Testing Behaviors**		
**No.**	**Question**	**Response**
5.	Have you ever been tested for active coronavirus infection? (nasal or saliva test)?	□ Yes<br>□ No
5a.	Have you ever tested positive for active coronavirus infection?	□ Yes<br>□ No
5a1.	When was your last positive test?	M M D D Y Y Y Y<br>□ I don't remember
5a2.	Have you experienced any long-term symptoms?	□ Yes<br>□ No
5a3.	Which of the following long-term symptoms did you experience?(please check all that apply)	□ Muscle weakness<br>□ Respiratory issues (e.g., shortness of breath, difficulty breathing, or persistent cough)<br>□ Lost or distorted senses of smell and/or taste<br>□ Neurological problems (e.g., fatigue, brain fog, or headaches)<br>□ Difficulty sleeping<br>□ Mental health issues (e.g., anxiety or depression)<br>□ Other (specify ___________________)<br>□ None of the above- I did not experience any long-term symptoms
5a4.	Overall, about how long did you experience symptoms for?	□ Two weeks<br>□ One month

	(including both short term and long term symptoms)	□ Two months<br>□ Three months or longer<br>□ Other (specify __________________)<br>□ None of the above- I was asymptomatic.
5b.	How often have you been tested for active coronavirus infection?	□ 1 time<br>□ 2-5 times<br>□ 6 or more times
5c.	Where have you gone for testing?<br>*Select all that apply.*	□ Doctor's office<br>□ Community pop-up or drive-up testing site<br>□ Other: ____
5d.	Why did you seek testing?	□ Close contact tested positive<br>□ Concern due to high cases in your community<br>□ Had coronavirus-*like* symptoms<br>□ Curious if you were positive<br>□ Other: ____
6.	Have you ever been tested for coronavirus antibodies (blood test)?	□ Yes<br>□ No
6a.	Have you ever tested <u>positive</u> for coronavirus antibodies?	□ Yes<br>□ No
6a1.	When was your last positive antibody test?	M | M | D | D | Y | Y | Y | Y<br>□ I don't remember
7.	Have you ever <u>NOT</u> sought testing for any reason?<br>Select all that apply.	□ No/Does not apply<br>□ Did not have transportation<br>□ Could not pay for the test<br>□ Inconvenient location<br>□ Inconvenient times and/or dates<br>□ Discomfort of the test<br>□ Other: ____
**Section 3: COVID-19 Vaccine**		
**No.**	**Question**	**Response**
8.	How do you feel about the following statements?	
8a.	I think the COVID-19 vaccines are safe	□ Agree<br>□ Neutral<br>□ Disagree
8b.	I think the COVID-19 vaccines are effective	□ Agree<br>□ Neutral<br>□ Disagree

8c.	I feel confident in the research process by the pharmaceutical companies that led to the design and development of the COVID-19 vaccines	□ Agree<br>□ Neutral<br>□ Disagree
8d.	I feel confident in the regulatory approval process by the US Food and Drug Administration (FDA) that led to the currently available COVID-19 vaccines on the market	□ Agree<br>□ Neutral<br>□ Disagree
9.	What are your motivations for taking the COVID-19 vaccine? (please check all that apply)	□ I do not plan to take the vaccine<br>□ Protecting a family or close friend that is high-risk for severe disease<br>□ Protecting myself<br>□ Concerned about possible virus exposures at work or school<br>□ Concerned about possible virus exposures in my community<br>□ Doing my part to help control the pandemic<br>□ Serving as an example to encourage others to take the vaccine
10.	What are possible barriers for taking the COVID-19 vaccine? (please check all that apply)	□ I do not believe I need it because I already had COVID-19 infection<br>□ Personal, religious, or cultural beliefs against COVID-19 vaccine<br>□ I am immunocompromised or pregnant<br>□ I do not think the vaccine is safe or effective<br>□ I do not know where to go to get the vaccine<br>□ I do not have time to take the vaccine<br>□ I do not have the money or transportation to get the vaccine<br>□ I have a fear of needles<br>□ I am not comfortable being one of the first people to get vaccinated
11.	Have you had the COVID-19 vaccine?	□ Yes<br>□ No
11A.	If yes, What as the date of your first vaccination?	(calendar or date formatted answer)

11A i	What as the date of your last vaccination?	(calendar or date formatted answer)
11A ii	Who was the manufacturer of your COVID-19 vaccine?	□ Pfizer<br>□ Moderna<br>□ Johnson and Johnson (Jenssen)<br>□ I do not know<br>□ Other
11B.	If no, Do you plan to take the COVID-19 vaccine?	□ Yes<br>□ No
11Bi.	If yes, when do you plan to take the COVID-19 vaccine?	□ In the next few weeks<br>□ I plan to wait for a while<br>□ Other (specify ___________________)
12.	Do you have any additional thoughts or comments regarding the COVID-19 vaccine?	(open ended)
**Section 4: Daily behaviors**		
**No.**	**Question**	**Response**
13.	In the past two weeks, how often have you practiced social distancing?	□ Never<br>□ Rarely<br>□ Some of the time<br>□ Most of the time<br>□ All the time
14.	In the past two weeks, how often do you wear a face covering when not at home?	□ Never<br>□ Rarely<br>□ Some of the time<br>□ Most of the time<br>□ All the time
15.	In the past two weeks, have you self-isolated or quarantined?	□ Yes<br>□ No
16.	In the past two weeks, how often have you felt stressed, nervous, anxious, or on edge?	□ Never<br>□ Rarely<br>□ Some of the time<br>□ Most of the time<br>□ All the time<br>□ Prefer not to answer
17.	In the past two weeks, how often have you felt sad, lonely, or depressed?	□ Never<br>□ Rarely<br>□ Some of the time<br>□ Most of the time<br>□ All the time<br>□ Prefer not to answer

18.	In the <u>past two weeks</u>, how often have you had physical reactions (e.g. *sweating, trouble breathing, nausea, or a pounding heart)* when thinking about your experience with the novel coronavirus (COVID-19) pandemic?	□ Never<br>□ Rarely<br>□ Some of the time<br>□ Most of the time<br>□ All the time<br>□ Prefer not to answer
19.	In the <u>past month</u>, were you or your family worried about not having enough food to eat due to a lack of money or other resources?	□ Yes<br>□ No<br>□ Prefer not to answer
20.	In the <u>past month</u>, did you or your family go without eating due to a lack of money or other resources?	□ Yes<br>□ No<br>□ Prefer not to answer
21.	What have you done to cope with your stress related to the COVID-19 outbreak? (please check all that apply)	□ Meditation and/or mindfulness practices<br>□ Talking with friends and family and/or engaging in more family activities<br>□ Increased television watching or other "screen time" activities (e.g., video games, social media)<br>□ Drinking alcohol<br>□ Using tobacco<br>□ Using marijuana<br>□ Talking to my healthcare providers more frequently, including mental healthcare provider (e.g., therapist, psychologist, counselor)<br>□ Other (specify __________________)
**Section 5: COVID-19 Risk Perception**		
**No**	**Question**	**Response**
21.	How concerned are you about yourself or someone in your household getting infected with coronavirus?	□ Not concerned<br>□ A little concerned<br>□ Concerned<br>□ Very concerned
22.	Have any of your close family or friends had or have coronavirus (COVID-19)?	□ Yes<br>□ No<br>□ Don't know
23.	How concerned are you about the spread of coronavirus (COVID-19) in your community <u>right now</u>?	□ Not concerned<br>□ A little concerned<br>□ Concerned<br>□ Very concerned
**Section 6: Parenting**		

No.	Question	Response
24	Are you a parent to a child under the age of 18 years?	Yes<br>No
24a	How many children under the age of 18 years live in your household?	____
24b	What are their ages	
24c	Do any of the following situations apply to your children?	□ None<br>□ Plan to put them in a summer camp<br>□ Has been enrolled in K-12 face-to-face since January 2021<br>□ Has been enrolled in K-12 hybrid since January 2021<br>□ Has been enrolled in K-12 virtual since January 2021<br>□ Has been attending daycare<br>□ Has been attending after school program or extra-curricular activities
25	Have any of your children been exposed to coronavirus infection at school/daycare/afterschool program?	□ Yes (describe exposure _______)<br>□ No
25a	If yes, how many notifications of potential COVID-19 exposure have you received in the 2020-2021 school year?	□ <5 times<br>□ 5-10 times<br>□ More than 10 times<br>□ Don't know
25b	Have any of your children had to quarantine at home due to a school/daycare/afterschool-related COVID-19 exposure?	□ Yes<br>□ No
27	Have any of your children ever tested <u>positive</u> for active coronavirus infection?	□ Yes (specify how many _______)<br>□ No
28	Do any children in your household have a high risk medical condition?	□ Yes (describe _______)<br>□ No
29	Regarding vaccination for your children, do any of these apply.	□ I plan to vaccinate my 6 month – 2 year old when eligible<br>□ I plan to vaccinate my 2-5 year old when eligible

	Select all that apply	□ I plan to vaccinate my 5-11 year old when eligible<br>□ I plan to vaccinate my 12-15 year old when eligible<br>□ I plan to vaccinate my 16+ year old
**Section 7: Almost done! Please tell us a little about yourself.**		
**No.**	**Question**	**Response**
30.	Do any of these situations apply to you?<br><br>*Select all that apply.*	□ None<br>□ I live in a nursing home, rehabilitation center, or long-term care facility<br>□ I work in a nursing home, rehabilitation center, or long-term care facility<br>□ I am a front-line medical care worker<br>□ I am an essential worker<br>(specify)______________________
31.	Do you have any health problems?<br><br>*Select all that apply.*	□ None<br>□ Asthma<br>□ Sickle cell disease<br>□ Lung disease (COPD, emphysema, etc)<br>□ Heart disease<br>□ Blood clotting disorder<br>□ Diabetes<br>□ High blood pressure (hypertension)<br>□ Immunocompromising condition (HIV, Lupus, cancer treatment, etc)<br>□ Other:<br>____
32.	In the past two weeks, have you experienced any of the following symptoms?<br><br>*Select all that apply.*	□ None, I feel healthy<br>□ Fever<br>□ Cough<br>□ Shortness of breath or difficulty breathing<br>□ Chest pain<br>□ Rapid heart rate or fast heartbeat<br>□ Muscle or body aches<br>□ Loss of taste<br>□ Loss of smell<br>□ Other<br>(specify)____________________________________
33.	How many people live in your home, including you?<br>*Please count anyone who sleeps in your home for 4 nights or more a week.*	[ ][ ]
34.	How tall are you?	___ feet ___inches

35.	How much do you weigh?	_____ lbs
36.	What is your age range?	□ <18 years old<br>□ 18-29 years old<br>□ 30-39 years old<br>□ 40-49 years old<br>□ 50-59 years old<br>□ 60-69 years old<br>□ 70+ years old
37.	What is(are) your race/ethnicity(ies)?<br><br>*Select all that apply.*	□ Hispanic/Latinx<br>□ White/Caucasian<br>□ Black/African American<br>□ Asian American<br>□ Native American/American Indian<br>□ Other (specify)_______________________________<br>□ Prefer not to say
38.	How do you define your gender?	□ Female<br>□ Male<br>□ Non-binary/third gender<br>□ Transgender<br>□ Prefer to self-describe<br>_________________________<br>□ Prefer not to say
39.	What was your household income last year?	□ Less than $15,000<br>□ $15,000 to $34,999<br>□ $35,000 to $49,999<br>□ $50,000 to $74,999<br>□ $75,000 to $99,999<br>□ $100,000 to $149,999<br>□ More than $150,000<br>□ Prefer not to say
**Section 8: Vaccine hesitancy, medical mistrust, and information sources. Again, this survey is anonymous.**		
**No.**	**Question**	**Response**

40.	How do you feel about the following statements?	
40a.	A lot of information about COVID-19 is being held back by the government.	□ Agree<br>□ Neutral<br>□ Disagree
40b.	The government cannot be trusted to tell the truth about COVID-19.	□ Agree<br>□ Neutral<br>□ Disagree
40c.	People should be suspicious of information from the government about COVID-19.	□ Agree<br>□ Neutral<br>□ Disagree
40d.	When it comes to COVID-19, people cannot trust health care providers.	□ Agree<br>□ Neutral<br>□ Disagree
40e.	When it comes to COVID-19, doctors have the best interests of patients in mind.	□ Agree<br>□ Neutral<br>□ Disagree
41.	Where did you get your information about COVID-19? (select all that apply)	□ Service providers or health professionals<br>□ News websites or apps<br>□ Announcements or news conferences held by local public health officials or agencies<br>□ Announcements or news conferences held by local government officials (e.g., a mayor or governor)<br>□ TV or radio<br>□ Social media<br>□ People I know, such as friends, family, neighbors, or coworkers<br>□ Briefings from the federal government, including the President of the United States<br>□ Social media<br>□ Other (specify)_______________________
